# The role of acetylation and deacetylation in cancer metabolism

**DOI:** 10.1002/ctm2.70145

**Published:** 2025-01-07

**Authors:** Cuicui Wang, Xiaoxin Ma

**Affiliations:** ^1^ Department of Obstetrics and Gynecology Shengjing Hospital of China Medical University Shenyang City Liaoning Province China; ^2^ Key Laboratory of Gynecological Oncology of Liaoning Province Department of Obstetrics and Gynecology Shengjing Hospital of China Medical University Shenyang Liaoning Province China

**Keywords:** acetylation, cancer, deacetylation, metabolic reprogramming

## Abstract

As a hallmark of cancer, metabolic reprogramming adjusts macromolecular synthesis, energy metabolism and redox homeostasis processes to adapt to and promote the complex biological processes of abnormal growth and proliferation. The complexity of metabolic reprogramming lies in its precise regulation by multiple levels and factors, including the interplay of multiple signalling pathways, precise regulation of transcription factors and dynamic adjustments in metabolic enzyme activity. In this complex regulatory network, acetylation and deacetylation, which are important post‐translational modifications, regulate key molecules and processes related to metabolic reprogramming by affecting protein function and stability. Dysregulation of acetylation and deacetylation may alter cancer cell metabolic patterns by affecting signalling pathways, transcription factors and metabolic enzyme activity related to metabolic reprogramming, increasing the susceptibility to rapid proliferation and survival. In this review, we focus on discussing how acetylation and deacetylation regulate cancer metabolism, thereby highlighting the central role of these post‐translational modifications in metabolic reprogramming, and hoping to provide strong support for the development of novel cancer treatment strategies.

**Key points:**

- Protein acetylation and deacetylation are key regulators of metabolic reprogramming in tumour cells.
- These modifications influence signalling pathways critical for tumour metabolism.
- They modulate the activity of transcription factors that drive gene expression changes.
- Metabolic enzymes are also affected, altering cellular metabolism to support tumour growth.

## Graphical abstract

Protein acetylation and deacetylation influence metabolic reprogramming in tumour cells by regulating the function and activity of various signalling pathways, transcription factors and metabolic enzymes.

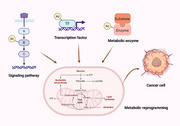

## 1 BACKGROUND

For cancer cells, metabolic changes occur not only to meet the demands of rapid proliferation but also to survive in the harsh tumour microenvironment.^1^ The characteristics of cancer metabolism are multifaceted, the most notable being the enhancement of macromolecular biosynthesis.^2^ Changes in energy metabolism are also important features of cancer metabolism. Cancer cells usually shift from relying on oxidative phosphorylation to glycolysis for energy, making them prefer glycolysis even in the presence of adequate oxygen, a phenomenon known as the ‘Warburg effect’.^3^ Moreover, the redox balance of cancer cells needs to be maintained to avoid damage from excessive reactive oxygen species (ROS) produced during metabolism.^4^ Therefore, by understanding the characteristics and mechanisms of cancer cell metabolism, we can develop targeted drugs for specific metabolic pathways to more effectively inhibit cancer cell growth and spread.^5^

Posttranslational modification of proteins is a key mechanism for regulating cell functions under specific conditions, allowing cells to adapt flexibly to environmental changes.^6^ As the major post‐translational modification of proteins, acetylation widely regulates the catalysis of multiple enzymes in cells.^7^ This finding suggested that the protein acetylation plays an important role in cellular metabolic cascades. Advanced mass spectrometry techniques have been able to comprehensively identify and describe thousands of acetylation sites, including lysine residues in cytoplasmic and nuclear proteins.^8^ These acetylation modifications profoundly affect cellular functions by regulating protein activity, subcellular localisation and stability.^8^ More critically, cancer cells adapt to changing microenvironments through protein acetylation modifications, dynamically regulating their phenotype through genetic and environmental factors.^9^, ^10^ This regulatory mechanism not only enables cancer cells to survive and proliferate in complex environments but also may affect the efficacy of therapeutic approaches.^9^, ^10^

For example, a deacetylase, histone deacetylase 4 (HDAC4) is up‐regulated in prostate cancer cell lines and regulates the expression of cell cycle‐related genes through deacetylation, thereby promoting tumour cell proliferation and malignant transformation.^11^ Sulforaphane from cruciferous vegetables may reduce prostate cancer risk by inhibiting HDAC6 and androgen receptor signalling.^12^ A protein isolated from bitter melon seeds, MCP30, can inhibit HDAC‐1 activity, promote the acetylation of histones H3 and H4, increase phosphatase and tensin homolog (PTEN) transcription, inhibit serine/threonine kinase B (Akt) phosphorylation and subsequently induce apoptosis in prostate intraepithelial neoplasia and prostate cancer cell lines.^13^ Additionally, HDACs are associated with the development of neurodegenerative and immune diseases.^14^ Therefore, the development of drugs targeting HDACs has become a research hotspot. Currently, several HDAC inhibitors, such as vorinostat, which has been approved for the treatment of cutaneous T‐cell lymphoma, have entered clinical trials.^15^ Other studies have shown that the deacetylase sirtuin 2 (SIRT2) can stabilise fibrinogen‐like protein 1 (FGL1) protein levels and that FGL1 plays a crucial role in tumour immune evasion. By using deacetylase inhibitors or aspirin, FGL1 protein acetylation can be increased, leading to its degradation and improving the efficacy of liver cancer immunotherapy.^16^ These findings provide a new strategy for the clinical treatment of liver cancer. At present, numerous approved and ongoing clinical trials focus on utilising acetylation mechanisms for cancer therapy (Table S1). It is believed that in the near future, more related drugs will be successfully applied in clinical translation.

This review discusses how acetylation and deacetylation regulate various signalling pathways, transcription factors and metabolic enzymes to understand how the protein acetylation affects metabolic reprogramming in tumour cells. This study provides a robust scientific basis for a deeper understanding of cancer cell survival strategies and the development of new cancer therapies.

## 2 CANCER METABOLISM

In the complex biological process of cancer, metabolic reprogramming is a crucial process. First, the abnormal hyperactivity of glucose metabolism provides a large energy source for cancer cells. Second, the enhancement of the fatty acid synthesis pathway ensures the stability of the cancer cell membrane structure and the supply of the raw materials needed for biosynthesis. Furthermore, the increase in glutamine decomposition also provides the necessary nitrogen and carbon sources for cancer cells, supporting their biosynthetic activities.^2^ In the regulatory network of tumour metabolic reprogramming, the abnormal activation of mechanistic target of rapamycin complex 1 (mTORC1) plays a pivotal role.^17^ This complex profoundly affects the metabolic state of cancer cells by influencing the synthesis of proteins, lipids and nucleotides.^18^ The PI3K (Phosphatidylinositol 3‐kinase)–AKT (Serine/Threonine Kinase B) signalling pathway is one of the important mechanisms for activating mTORC1. It ultimately leads to the activation of mTORC1 through a series of signal transductions, thereby promoting the metabolic reprogramming of cancer cells.^19^ In addition to the PI3K–AKT signalling pathway, abnormal activation of the MAPK (Mitogen‐activated Protein Kinase) and JAK/STAT3 (Janus Kinase/Signal Transducer and Activator of Transcription 3) signalling pathways also plays a key role in metabolic reprogramming.^20^, ^21^ Abnormal activation of these signalling pathways further exacerbates metabolic abnormalities in cancer cells by affecting the expression of various metabolic enzymes and transcription factors within the cells.

Metabolic enzymes are direct targets of growth factor signalling cascades and oncogenic transcription factors.^22^ Early studies revealed that the oncogene ras can induce the expression of glucose transporters, enabling cancer cells to take up large amounts of glucose to meet their abnormal energy demands.^23^ Subsequently, the oncogenic transcription factor c‐myc can directly activate lactate dehydrogenase A (LDHA). LDHA is a key metabolic enzyme that converts pyruvate, produced during glucose metabolism, into lactate. This conversion process not only provides an additional energy source for cancer cells but also promotes the growth and metastasis of cancer cells by affecting the acid‒base balance of the intracellular environment.^24^ Studies have also shown that LDHA is necessary for the transformation induced by c‐myc in cancer cells. Cancer cells growing in a three‐dimensional environment tend to be more invasive and metastatic, and the activation of LDHA is crucial for these cancer cells to achieve this transformation.^24^ As metabolic reprogramming progresses, a large amount of ROS are produced inside cancer cells.^25^ The accumulation of these ROS not only threatens the survival of cancer cells but also affects the malignant behaviour of cancer cells through a series of signalling pathways. To cope with this challenge, cancer cells enhance their antioxidant capacity by adjusting the expression of transcription factors such as NRF2 (Nuclear factor erythroid‐2‐related factor‐2) and HIF‐1 (Hypoxia inducible factor‐1) to ensure survival under ROS stress.^26^

## 3 ACETYLATION AND DEACETYLATION

**FIGURE 1:**
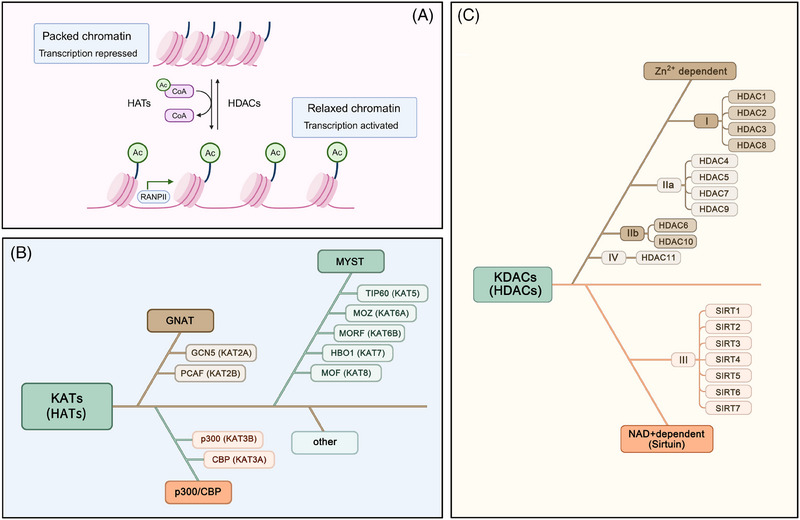
Modulation of the reversible process of lysine acetylation. (A) Schematic diagram of the reversible process of histone modifications created by Biorender. In the green circle, Ac represents the acetyl group. In the purple circle, CoA represents coenzyme A. (B) Classification of acetyltransferases. The remaining KATs, which are classified as ‘other’, exhibit relatively minor dissimilarities among themselves, and research on this particular aspect has been relatively scarce. (C) Classification of deacetylases. Lysine deacetylases (KDACs) can be classified into two primary classes: classical HDACs, which rely on Zn2+ for their activity, and sirtuin deacetylases, which are dependent on NAD+. Within these broader categories, KDACs can be further subcategorised into Class I, Class IIa, Class IIb, Class III and Class IV. CBP, CREB‐binding protein; GCN5, general control of amino acid synthesis protein 5; GNAT, GCN5‐related N‐acetyltransferases; HATs, histone acetyltransferases; HBO1, histone acetyltransferase binding to ORC1; HDACs, histone deacetylases; KATs, lysine acetyltransferases; KDACs, lysine deacetylases; MOF, males absent on the first; MORF, monocytic leukemic zinc finger‐related factor; MOZ, monocytic leukaemia zinc finger protein; MYST, MOZ/Ybf2 (Sas3)/Sas2/Tip60; NAD, nicotinamide adenine dinucleotide; PCAF, p300/CBP‐associated factor; RNAPII, RNA polymerase II; SIRT, sirtuin; Tip60, 60 kDa tat‐interactive protein.

Acetylation refers to the process in which acetyl groups are transferred from acetyl‐CoA to protein amino acid residues under the catalysis of specific enzymes (such as acetyltransferases). Acetylation modifications are mainly divided into the N‐terminal proteins acetylation and acetylation, which are the ε‐amino side chains of lysine residues on proteins.^27^ Among these modifications, the acetylation modification of lysine residues in proteins is a dynamic and reversible process that is the main form of acetylation and is controlled by the coordinated action of lysine acetyltransferases (KATs) and lysine deacetylases (KDACs).^28^ Histone acetylation refers to the addition of acetyl groups to lysine residues on histones by histone acetyltransferases. This reduces the positive charge of histones, decreasing their affinity for negatively charged DNA molecules and leading to the relaxation of nucleosome structure, thereby promoting the specific binding of various transcription factors to DNA‐binding sites and activating gene transcription^7^ (Figure 1A). Non‐histone acetylation pertains to the acetylation processes that take place on proteins located within the cytoplasm and various other subcellular compartments. This modification influences protein functionality via multiple mechanisms, such as modulating protein stability, altering enzyme activity, determining subcellular localisation and interacting with other post‐translational modifications.^29^ KATs are mainly divided into three families: (1) the GNAT family, comprising GCN5 (KAT2A) and PCAF (KAT2B); (2) the p300/CBP family, which encompasses CBP (KAT3A) and p300 (KAT3B); and (3) the MYST family, which consists of TIP60 (KAT5), MOZ (KAT6A), MORF (KAT6B), HBO1 (KAT7) and MOF (KAT8)^30^ (Figure 1B). Deacetylases are a group of proteins known as KDACs or HDACs. According to enzyme structure, function and cofactor dependency, HDACs can be classified into four primary categories: Class I comprises HDAC1, HDAC2, HDAC3 and HDAC8; Class II HDACs are further subdivided into two subclasses: Class IIa and Class IIb. The Class IIa subclass includes HDAC4, HDAC5, HDAC7 and HDAC9, whereas HDAC6 and HDAC10 are categorised under Class IIb; Class III HDACs (also known as the sirtuins family) include SIRT1 to SIRT7; and Class IV HDACs generally refer to HDAC11.^30^ The deacetylase activity of the sirtuin family depends on nicotinamide adenine dinucleotide (NAD+).^31^ The other three classes of HDACs are Zn2+‐dependent enzymes^32^ (Figure 1C). Acetyltransferases and deacetylases play crucial roles in gene expression regulation, protein function and cellular signalling and are essential for cell growth, differentiation, metabolism and survival.

### 3.1 Acetylation and the PI3K/AKT/mTOR signalling pathway

The PI3K/AKT/mTOR signalling pathway plays a central regulatory role in cell metabolism, growth, proliferation and survival.^33^ When growth factors bind to receptor tyrosine kinases (RTKs), the receptors are activated, which in turn promotes the activation of PI3K. PI3K phosphorylates PIP2 (Phosphatidylinositol 4,5‐bisphosphate) to PIP3 (Phosphatidylinositol 3,4,5‐trisphosphate). This process facilitates the recruitment of the kinases PDK1 and AKT to the cellular membrane, which subsequently results in the activation of AKT through a PDK1‐dependent mechanism. The activation of AKT further triggers the activation of downstream mTORC1 complexes, which play critical roles in cell growth and metabolism.^34^ In cancer cells, RTKs significantly up‐regulate glucose uptake and glycolysis via the PI3K/Akt/mTOR signalling pathway, leading to the production of a large amount of pyruvate, which is converted into lactate or enters the mitochondria for other metabolic pathways.^35^

#### 3.1.1 Acetylation of PI3K

**FIGURE 2:**
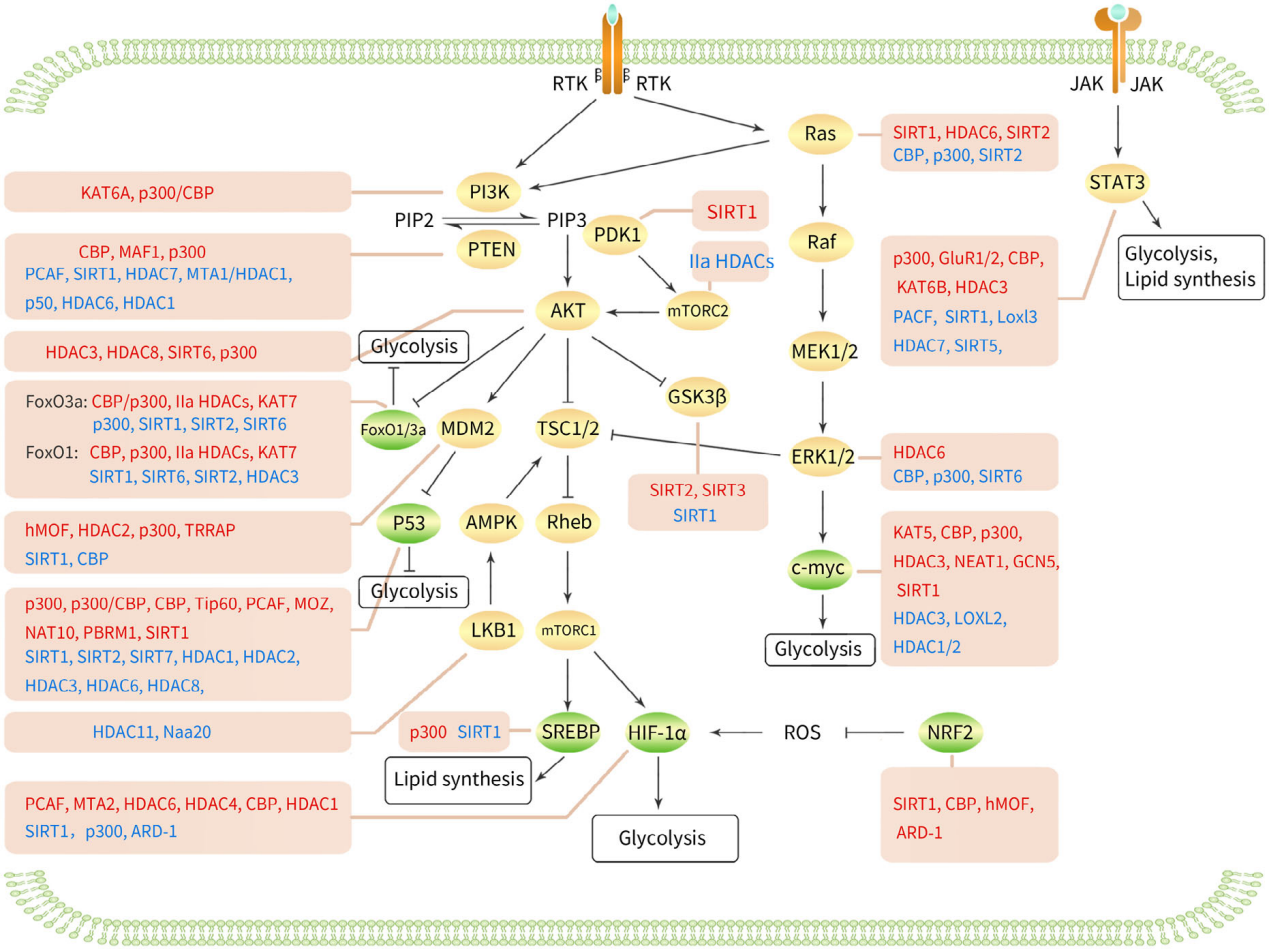
Regulation of signalling pathways and transcription factors related to cancer metabolism through acetylation and deacetylation. Red text indicates the positive regulatory relationship of acetyltransferases and deacetylase on protein activity or expression levels. Blue text indicates the negative regulatory relationship of acetyltransferases and deacetylase on protein activity or expression levels. AKT, serine/threonine kinase B; AMPK, AMP‐activated protein kinase; ARD‐1, antisense arrest‐defective 1 protein; c‐myc, cellular myelocytomatosis oncogene; ERK1/2, extracellular signal‐regulated kinase 1/2; FOXOs, forkhead box class O proteins; GluR, glutamate receptors; GSK3β, glycogen synthase kinase‐3 beta; HIF‐1α, hypoxia inducible factor‐1α; JAK, Janus kinase; LKB1, liver kinase B1; Loxl3, LOX‐like 3; MDM2, mouse double minute 2 homolog; MEK1/2, MAPK/ERK kinase1/2; MTA, metastasis‐associated protein; mTORC, mechanistic target of rapamycin complex; Naa20, N‐α‐acetyltransferase 20; NAT10, N‐acetyltransferase 10; NRF2, nuclear factor erythroid‐2‐related factor‐2; PBRM1, polybromo‐1; PDK1, 3‐phosphoinositide‐dependent protein kinase 1; PI3K, phosphatidylinositol 3‐kinase; PIP2, phosphatidylinositol 4,5‐bisphosphate; PIP3, phosphatidylinositol 3,4,5‐trisphosphate; PTEN, phosphatase and tensin homolog; Ras, rat sarcoma; ROS, reactive oxygen species; RTK1 receptor tyrosine kinase; SREBP, sterol‐regulatory element binding proteins; STAT3, signal transducer and activator of transcription 3; TSC1/2, tuberous sclerosis complex1/2.

The p110α protein encoded by the PIK3CA gene is a catalytic subunit of the PI3K enzyme. This subunit works in conjunction with other PI3K subunits encoded by different genes to regulate the activity of the PI3K enzyme.^36^ KAT6A is responsible for the acetylation of lysine 23 on histone H3, which facilitates the recruitment of the nuclear receptor coactivator TRIM24. This interaction leads to the activation of PIK3CA transcription, consequently promoting the PI3K/AKT signalling cascade and contributing to the progression of glioblastoma.^36^ The actin‐binding protein CapG interacts with p300/CBP and binds to the specific promoter of the regulatory subunit PIK3R1/P50 of PI3K, increasing PIK3R1/P50 transcription by acetylating lysine 27 of histone H3, thereby activating PI3K/Akt and conferring paclitaxel resistance in breast cancer patients^37^ (Figure 2).

#### 3.1.2 Acetylation of PTEN

PTEN is a tumour suppressor gene located on human chromosome 10q23 that can dephosphorylate PIP3 to PIP2, thereby negatively regulating the PI3K/AKT pathway and inhibiting tumour cell growth.^38^ Moreover, PCAF acetylates lysine 125 and 128 in the active site of PTEN, weakening its ability to down‐regulate PI3K signalling and induce G1 cell cycle arrest, which promotes tumour formation and progression.^39^ CBP acetylates lysine 402 of PTEN, facilitating its interaction with PDZ domain‐containing proteins. Conversely, SIRT1 deacetylates this site, inhibiting the interaction of PTEN with PDZ domain‐containing proteins, thus influencing the development of prostate cancer.^40^ These observations indicate that different acetyltransferases can acetylate the same protein, PTEN, at different lysine residues.

MAF1 can inhibit the transcription of ribosomal and transfer RNA genes, which are regulated by the mTOR signalling pathway. MAF1 enhances acetylation and the transcription of the PTEN promoter by binding to it, inhibiting the AKT–mTOR signalling pathway in liver cancer.^41^ P300 can synergise with EGR1 to acetylate the core histones of PTEN and induce its transcription, thereby enhancing doxorubicin‐induced tumour cell apoptosis.^42^ In liver cancer stem cells, miR‐342‐3p targets HDAC7, promoting histone H3 acetylation and PTEN transcription, thereby inhibiting stem cell tumourigenicity and stemness.^43^ Pterostilbene (PTER) has been shown to have anti‐tumour effects on hepatocellular carcinoma (HCC). PTER disrupts the MTA1/HDAC1 complex, acetylates PTEN at lysine 402 and activates PTEN, thereby promoting apoptosis and inhibiting HCC growth and invasion.^44^ Prolonged in vitro exposure to monomethylarsenous acid (MMA(III)) can lead to bladder cancer. MMA(III) activates NF‐κβ p50 homodimers and inhibits the H3 acetylation in the PTEN promoter, leading to chromatin remodelling around the PTEN promoter and reducing PTEN expression, thus accelerating malignant cell transformation.^45^ PTEN activation depends on membrane translocation. HDAC6 deacetylates PTEN at K163, promoting interaction between the PTEN C‐terminus and the rest of the protein and inhibiting its membrane translocation, thus inactivating PTEN and promoting tumour growth.^46^ Epidemiological studies have shown that a protein isolated from bitter melon seeds, MCP30, can inhibit HDAC1 activity, promote histone H3 and H4 acetylation, increase PTEN transcription and inhibit AKT phosphorylation, thereby inducing apoptosis in prostate intraepithelial neoplasia and prostate cancer cell lines.^13^

#### 3.1.3 Acetylation of PD

AKT and PDK1 are acetylated at lysine residues enriched in pleckstrin homology domains, as these domains mediate the binding of PIP3 to AKT and PDK1, and acetylated AKT and PDK1 prevent their binding to PIP3, thereby preventing AKT membrane localisation and phosphorylation. Conversely, SIRT1 promotes the binding of AKT and PDK1 to PIP3 by deacetylating them, thereby activating AKT signalling and tumour growth.^47^

#### 3.1.4 Acetylation of AKT

Polyubiquitination is a key step in growth factor‐induced AKT membrane localisation and phosphorylation.^48^ Under the stimulation of growth factors, HDAC3 interacts with the scaffold protein APPL to promote deacetylation of AKT at lysines 14 and 20, leading to polyubiquitination and phosphorylation of AKT, activation of the AKT–mTOR signalling pathway, and promotion of prostate cancer cell growth.^49^ In leukaemia cells, chemotherapeutic drugs up‐regulate HDAC3 expression, and HDAC3 deacetylates AKT at K20, thereby promoting AKT phosphorylation, accelerating leukaemia progression and inducing chemotherapeutic resistance in MLL‐AF9(+) leukaemia cells.^50^ In breast cancer tissues and cells, HDAC8 expression is up‐regulated, and HDAC8 interacts with AKT1, deacetylating it at lysine 426 and activating AKT1, thereby increasing GSK‐3β phosphorylation and promoting its degradation, which triggers the spread and EMT of breast cancer cells.^51^ In HCC, high levels of SIRT6 deacetylate AKT, leading to increased AKT phosphorylation and activity. Activated AKT then phosphorylates the anti‐apoptotic protein XIAP at Ser87, increasing its protein stability to maintain the oncogenic functions of tumour cells.^52^ Interleukin (IL)‐17A is a cytokine secreted by T helper 17 cells. In the tumour microenvironment of human nasopharyngeal carcinoma, IL‐17A mediates AKT1 acetylation via p300, activating the Akt signalling pathway and stimulating the proliferation of nasopharyngeal carcinoma cells.^53^

#### 3.1.5 Acetylation of mTOR

mTORC2 is one of the two forms of the mammalian target of rapamycin complex, and together with mTORC1, it forms two functionally and structurally distinct complexes of mTOR.^54^ mTORC2 fully activates AKT enzyme activity by phosphorylating the Ser473 site of AKT serine, thereby promoting cell proliferation and inhibiting apoptosis.^55^ Rictor is the core component of the mTORC2 signalling complex. Elevated glucose levels can acetylate Rictor in an acetyl‐CoA‐dependent manner through the glycolytic pathway, and acetylated Rictor can maintain mTORC2 self‐activation, thereby reducing the sensitivity of glioblastoma cells to PI3K/AKT‐targeted therapy. Class IIa HDACs can deacetylate Rictor and inhibit the self‐activation loop of mTORC2.^56^

#### 3.1.6 Acetylation of MDM2

MDM2 (mouse double minute 2 homolog) is an E3 ubiquitin ligase that can inhibit the transcriptional activity of p53 and mediate the ubiquitination and degradation of p53 after entering the nucleus, indirectly affecting the stability of p53.^57^, ^58^ Activation of AKT can mediate the entry of MDM2 into the nucleus, regulate the expression of p53 and affect cell proliferation and apoptosis.^59^ MDM2, a novel non‐histone substrate of hMOF, directly interacts with hMOF, leading to MDM2 acetylation, which inhibits its ubiquitination and degradation, thereby increasing MDM2 stability and enhancing cisplatin resistance in ovarian cancer cells.^60^ HDAC2 deacetylates MDM2, promoting the recognition and degradation of its downstream substrate MCL‐1 ubiquitin ligase (MULE), reducing the degradation of the fusion product SS18‐SSX and thereby promoting the occurrence of t(X;18) translocation‐associated synovial sarcoma.^61^ After P300 acetylates MDM2 at lysine residues 182 and 185, it stabilises MDM2 by binding to HAUSP and increasing p53 ubiquitination. On the other hand, SIRT1 deacetylates MDM2 at lysine residues 182 and 185, promoting its own ubiquitination, increasing p53 stability and inducing apoptosis in tumour cells.^62^ MDM2 can also be acetylated by CBP in vitro, with acetylation sites primarily occurring in the RING finger domain of MDM2. CBP‐mediated acetylation of MDM2 inactivates MDM2 and inhibits the degradation of p53.^63^ Interestingly, MDM2 is also a transcriptional target gene of p53. TRRAP is a component of several acetyltransferase complexes, and when p53 binds to TRRAP, it can be recruited to the MDM2 promoter region, increasing histone acetylation and activating MDM2 transcription, thereby enhancing p53 proteasomal degradation and forming a negative feedback loop.^64^

#### 3.1.7 Acetylation of LKB1

AMP‐activated protein kinase (AMPK) is a key molecule in the regulation of bioenergy metabolism and the primary sensor of cellular energy status.^65^ AMPK can inhibit the activity of mTORC1 by activating the upstream inhibitors TSC1/2 of mTORC1.^66^ Liver kinase B1 (LKB1), the main upstream kinase of AMPK, is a serine/threonine kinase that can directly phosphorylate the α subunit of AMPK to activate the AMPK signalling pathway. Therefore, LKB1 can inhibit mTORC1 activity by activating AMPK, thereby regulating a series of energy metabolism‐related processes.^67^ HDAC11 expression is elevated in HCC, where it inhibits the transcription of LKB1 by suppressing histone acetylation in the promoter region of LKB1, thereby silencing the AMPK signalling pathway and activating mTORC1 activity and glycolysis, leading to stem cell properties and the progression of HCC.^68^ N‐α‐acetyltransferase 20 (Naa20) is the catalytic subunit of the N‐terminal acetyltransferase B complex, and its expression is also increased in HCC. Naa20 inhibits the LKB1–AMPK signalling pathway via the N‐terminal acetylation of LKB1, thereby promoting cell proliferation and autophagy.^69^

#### 3.1.8 Acetylation of GSK3β

Glycogen synthase kinase 3β (GSK3β) is a highly conserved serine/threonine kinase that is widely present in mammalian eukaryotic cells. It participates in glycogen synthesis by regulating the activity of glycogen synthase.^70^ AKT can reduce the activity of GSK3β by phosphorylating the Ser9 site of GSK3β, thereby promoting glucose uptake and utilisation in tumour cells and increasing the energy supply of tumour cells.^71^ SITR1 cooperates with the activity regulator AROS to inhibit the acetylation of GSK3β, inactivating GSK3β, which weakens doxorubicin‐mediated apoptosis in neuroblastoma treatment, leading to tumour resistance.^72^ In breast cancer, SIRT2 inhibits the acetylation of GSK3β in CD8(+) effector memory T cells, leading to an increase in the number of effector memory T cells, enhancing the tumour immune response and slowing tumour progression.^73^ SITR3 can also activate GSK3β by mediating the deacetylation of GSK3β. In NHL and HCC, SIRT3 deacetylates and activates GSK‐3β, inducing the expression and mitochondrial translocation of the proapoptotic protein Bax and triggering mitochondrial apoptosis, thereby inhibiting tumour growth.^74^, ^75^

### 3.2 Acetylation and the MAPK signalling pathway

The MAPK signalling pathway is an important signal transduction system in organisms that transmits signals through a three‐tier kinase cascade.^76^ The MAPK family mainly includes four subfamilies, ERK1/2, JNK, p38 and ERK5/BMK1, which represent four classical MAPK pathways. Among these pathways, the RAS–Raf–MEK–ERK signalling pathway is the most extensively studied and is most closely related to tumours.^77^ Activation of the ERK signalling pathway can promote tumour cell proliferation and metabolism by increasing c‐myc transcription.^78^

#### 3.2.1 Acetylation of KRAS

KRAS belongs to the RAS gene family and encodes a protein that is anchored to the membrane. It plays a crucial role as an integral part of the MAPK signalling pathway and functions as an upstream activator protein.^77^ CBP can directly acetylate KRAS, and acetylated KRAS inhibits RAS/RAF/MEK/ERK signal transduction in acute lymphoblastic leukaemia cells with Ras pathway mutations.^79^ Acetylation of RAS at lysine 104 interferes with guanine nucleotide exchange factor‐induced nucleotide exchange, inhibiting the transforming activity of KRAS and thereby reducing cell proliferation, colony formation and tumour burden in mice.^80^ In non‐small cell lung cancer, p300 acetylates KRAS at lysine 104, while SIRT1 can deacetylate this site.^81^, ^82^ HDAC6 and SIRT2 can also deacetylate KRAS at lysine 104 and promote cancer cell growth.^83^ Another study indicated that SIRT2 can also deacetylate KRAS at K147 and that its acetylation status directly regulates KRAS activity, ultimately inhibiting tumour growth and invasion.^84^ Thus, even the same acetyltransferase or deacetylase, by acetylating different lysine sites on a protein, may result in entirely different biological effects.

#### 3.2.2 Acetylation of ERK1/2

ERK (extracellular regulated protein kinase) is a subfamily of MAPKs that includes two isoforms, ERK1 and ERK2, which are key for transmitting signals from surface receptors to the nucleus.^85^ Both CBP and p300 can acetylate ERK1 at the N‐terminal K72 site, and HDAC6 deacetylates ERK1/2. The transcription factor ELK1 is a substrate of ERK1, deacetylated ERK1 activates ERK1 activity, increasing ELK1 enzyme activity and thereby promoting tumour cell proliferation, migration and invasion.^86^ In multiple myeloma, SIRT6 down‐regulates ERK1 transcription by deacetylating H3K9 in the ERK1 promoter region, thereby inhibiting MAPK signalling and proliferation. Additionally, high levels of SIRT6 attenuate ERK/p90RSK signalling and enhance Chk1‐mediated DNA repair, thus regulating the DNA damage response in tumour cells.^87^

### 3.3 Acetylation and the JAK/STAT3 signalling pathway

The JAK/STAT3 signalling pathway is a specific pathway within the JAK–STAT signalling cascade, with STAT3 being a key transcription factor in this pathway. JAKs are a class of non‐RTKs that, upon binding to cytokine receptors, phosphorylate specific tyrosine residues on STAT3, allowing it to form dimers and translocate into the nucleus to regulate the transcription of target genes.^88^ STAT3 plays a critical role in glucose metabolism by regulating the expression of genes involved in glycolysis and oxidative phosphorylation, thereby affecting the energy metabolism of tumours.^89^ STAT3 is also involved in regulating lipid metabolism in tumour cells, affecting lipid synthesis, breakdown and uptake.^90^ In HCC, activation of the IL‐6/STAT3 pathway directly enhances the expression of TIMP‐1 in liver cancer cells, converting normal hepatic fibroblasts to cancer‐associated fibroblasts, thus altering the tumour microenvironment and promoting tumourigenesis. PCAFA directly acetylates STAT3, negatively regulating TIMP‐1 expression by inhibiting the IL‐6/STAT3 pathway, thereby suppressing the growth of HCC.^91^ P300 can mediate STAT3 acetylation in various malignancies, such as prostate cancer, HCC and breast cancer.^92^, ^93^, ^94^, ^95^ In prostate cancer, p300‐mediated acetylation of STAT3 at the K685 site stabilises STAT3 dimers. Stabilised STAT3 increases the transcription of genes related to tumour cell growth and cell cycle progression.^92^, ^96^ When the glutamate receptors GluR1 and GluR2 are acetylated by CBP, they can directly acetylate the lysine 685 site of STAT3 by recruiting β‐arrestin 1/2, leading to its mitochondrial translocation and increased transcription of energy metabolism‐related genes, thereby inducing cell proliferation.^97^ SITR1 is an NAD+‐dependent deacetylase that deacetylates STAT3, reducing its phosphorylation and inhibiting its activity.^98^, ^99^ Supplementing NAD precursors, such as nicotinamide or niacin, can inactivate STAT3 and reverse tumour epithelial–mesenchymal transition (EMT), with effects comparable to those of STAT3 inhibitors.^100^ IL‐6 can promote the binding of SIRT1 to JAK1, leading to SIRT1 phosphorylation. Phosphorylated SIRT1 reduces STAT3 acetylation and transcriptional activity.^101^ Loxl3 is a member of the lysyl oxidase (LOX) family with protein deacetylation functions. After binding to STAT3 in the nucleus, Loxl3 deacetylates STAT3, disrupting its dimerisation and weakening its transcriptional activity and cell proliferation.^102^ In addition, HDAC7 and SIRT5 can also reduce STAT3 acetylation levels, promoting tumour apoptosis,^103^, ^104^ whereas CBP and KAT6B, as acetyltransferases, can reverse this process.^104^, ^105^, ^106^ Notably, in multiple myeloma, although HDAC3 reduces STAT3 acetylation levels, it also promotes the phosphorylation of STAT3 at tyrosine 705 and serine 727, thereby significantly inhibiting tumour cell apoptosis and promoting cell growth, indicating possible crosstalk between acetylation and phosphorylation.^107^

### 3.4 Acetylation and transcription factors

#### 3.4.1 Acetylation of FoxO1/3a

**FIGURE 3:**
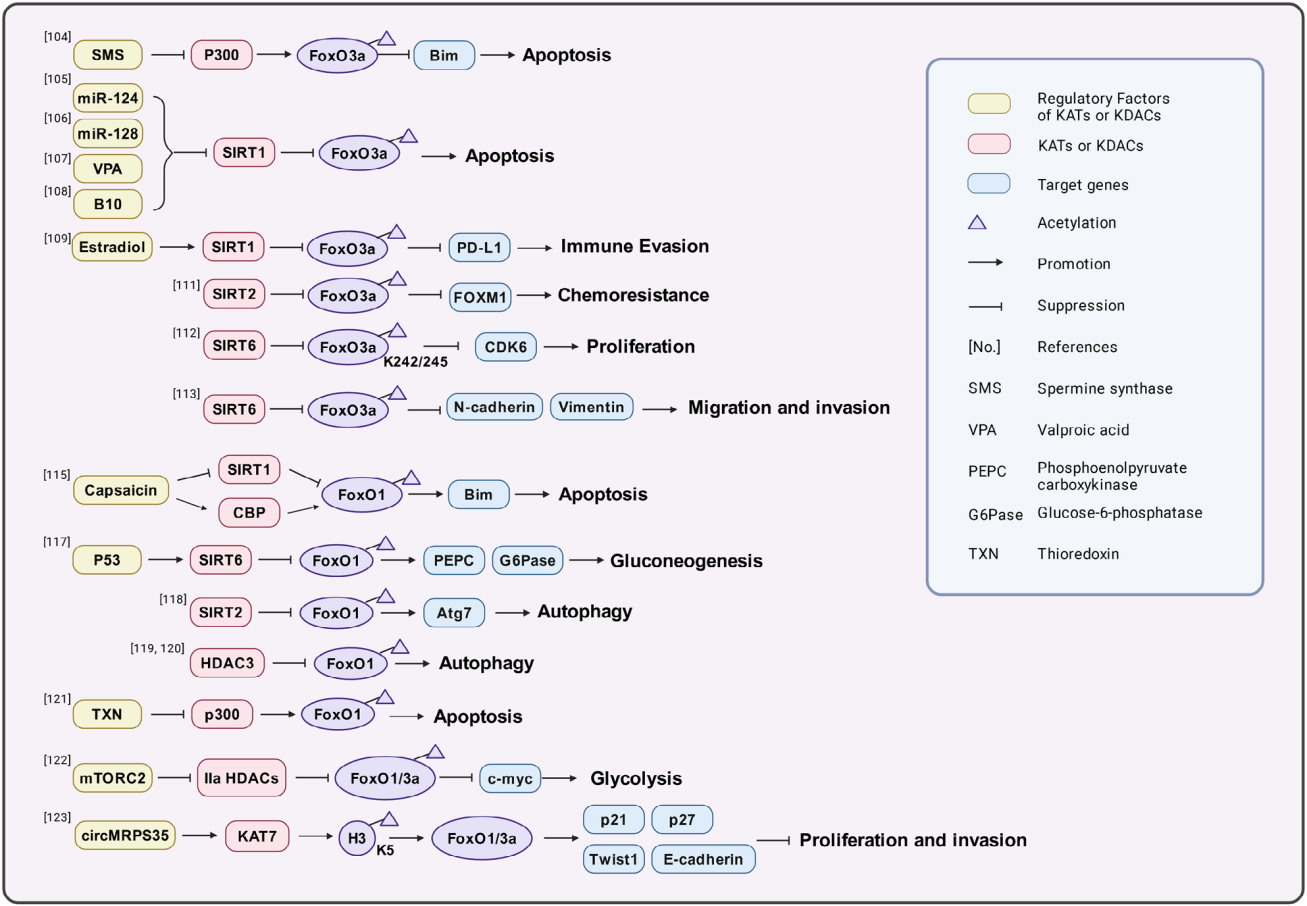
Summary of the mechanisms of FoxO1/3a acetylation in cancer.

FoxO1/3a are two proteins in the FOXO family. As transcription factors, they regulate the expression of glycolysis‐related genes, including key enzymes in the glycolytic pathway, such as hexokinase, 6‐phosphofructokinase and pyruvate kinase, reducing their activity and thus inhibiting glycolysis.^108^ AKT phosphorylates specific amino acid sites of FoxO1/3a, causing them to translocate from the nucleus to the cytoplasm. This phosphorylation process reduces the transcriptional activity of FOXO in the nucleus, thereby affecting its expression.^108^, ^109^ Spermine synthase converts spermidine into spermine. In colorectal cancer cells, targeting spermine synthase leads to spermidine accumulation and inhibits p300‐mediated acetylation of FoxO3a, causing FoxO3a to translocate to the nucleus and inducing the expression of the apoptosis protein Bim, thereby reducing tumour size^110^ (Figure 3). Inhibiting SIRT1 can maintain the acetylation level of FoxO3a, inducing the apoptosis of tumour cells^111^, ^112^, ^113^, ^114^ or inhibiting immune evasion.^115^ In prostate cancer cells, SIRT1/2 also promotes the multiubiquitination and proteasomal degradation of FoxO3 mediated by the E3 ubiquitin ligase subunit Skp2 by deacetylating FoxO3, thereby down‐regulating FoxO3 protein levels.^116^ In nasopharyngeal carcinoma, SIRT2 mediates the deacetylation of FoxO3, resulting in FoxO3 inactivation. Inactivated FoxO3 promotes the expression of its target gene FOXM1, thereby increasing resistance to lapatinib.^117^ In breast cancer cells, disrupting the interaction between SIRT6 and FoxO3a leads to FoxO3a acetylation. Acetylation at the K242/245 sites promote the binding of FoxO3a to BRD4 and induces the transcription of the CDK6 gene in the cyclin‐dependent kinase family.^118^ In HCC, SIRT6 increases the expression of N‐cadherin and vimentin by deacetylating FoxO3a, promoting cell migration and invasion.^119^ In addition to SIRT6, SIRT1/2 and CBP/p300 can also alter the acetylation levels at the K242/245 sites of FoxO3a in B‐ALL.^120^ Capsaicin, a pungent alkaloid compound, increases FoxO1 acetylation levels by up‐regulating CBP expression and down‐regulating SIRT1 expression in pancreatic cancer cells. This stabilises nuclear FoxO1 expression, thereby increasing Bim transcription and promoting apoptosis.^121^, ^122^ FoxO1 can also be deacetylated by SIRT6, and under the direct influence of p53, the expression of SIRT6 increases, causing deacetylated FoxO1 to be exported to the cytoplasm. This leads to a decrease in the expression of key enzymes that are critical for gluconeogenesis, including phosphoenolpyruvate carboxykinase (PEPCK) and glucose‐6‐phosphatase (G6Pase), decreasing the production of glucose from non‐sugar precursors and inhibiting tumour cell growth.^123^ SIRT2 and HDAC3 can also mediate the deacetylation of FOXO1, inhibiting FOXO1 activity and autophagy and thereby promoting cancer progression.^124^, ^125^, ^126^ Thioredoxin (TXN) is a class of small redox proteins widely present in organisms that are involved in the transmission of redox signals. In diffuse large B‐cell lymphoma, knocking out TXN can promote p300‐mediated acetylation of FoxO1 and expression of FoxO1, thereby increasing FoxO1 transcription of apoptosis genes and cell cycle inhibitory genes.^127^ mTORC2 promotes the inactivation of Class IIa HDACs, which in turn acetylate FoxO1 and FoxO3a, thereby relieving the transcriptional repression of FOXO on c‐myc and promoting aerobic glycolysis in glioblastoma.^128^ circMRPS35 functions as a scaffold that facilitates the recruitment of KAT7 to the promoters of the FOXO1/3a genes. This interaction promotes the acetylation of H4K5, which in turn activates the transcription of FOXO1/3a. The subsequent expression of downstream tumour suppressor genes, including p21, p27, Twist1 and E‐cadherin, leads to the inhibition of proliferation and invasion in gastric cancer cells.^129^

#### 3.4.2 Acetylation of p53

P53 is a well‐known tumour suppressor protein, also known as the TP53 or p53 protein, which plays a crucial role in various biological processes, such as cell cycle regulation, DNA damage repair, apoptosis and aging.^130^, ^131^ In malignant tumours, the p53 gene frequently mutates, leading to the loss of p53 protein function, reducing the inhibition of glycolysis, enhancing the energy supply of tumour cells and thereby promoting tumour growth.^132^ p53 is the first non‐histone protein proven to be acetylated.^133^ P300 and CBP are the first acetyltransferases found to directly bind to the p53 protein. In various malignancies, P300 and CBP can acetylate multiple lysine residues on p53, and acetylated p53 significantly enhances DNA binding ability and transcriptional activity, thereby promoting tumour cell cycle arrest, senescence and apoptosis (Table 1). SIRT1 is the most studied p53 deacetylase to date, and in most cases, SIRT1 mediates the deacetylation of p53, weakening its transcriptional regulatory ability. SIRT2, SIRT7, HDAC1, HDAC2, HDAC3, HDAC6 and HDAC8 can also reduce the acetylation levels of p53, inhibiting cell apoptosis and thereby promoting tumour growth (Table 2).

**TABLE 1:** Acetyltransferases of p53.

Acetyltransferase	Cancer type	Acetylation site	Function	Regulatory factor^a^	References
p300	High‐grade serous ovarian carcinoma	N/A	P300‐mediated acetylation of p53 increases p53 stability and transcriptional activity, inducing cellular senescence.	N/A	^134^
	N/A	N/A	Activated p300 stimulates p53 acetylation and promotes tumour cell apoptosis.	TSPYL2 &	^135^
	B‐cell lymphoma	K132	Inhibited p300 suppresses p53 acetylation, reducing p53‐dependent caspase‐1 transcription.	BCL6 #	^136^
	N/A	N/A	Skp2 prevents p53 acetylation‐mediated tumour cell apoptosis by competitively binding with p300.	Skp2 #	^137^
	Colorectal cancer	K382	Activated p300 acetylates and activates p53.	ArhGAP30 &	^138^
	Prostate cancer, breast cancer, cervical cancer	N/A	By interacting with p53, the acetylation process of p53 by p300 is inhibited, thereby weakening the ability of p53 to bind to DNA elements distant from its binding sites.	BOZF1 #	^139^
	Ewing family tumours	K382	By directing p300 to acetylate p53, the transcriptional activity and protein stability of p53 are enhanced.	EWS‐Fli1 #	^140^
p300/CBP	Stomach/rectal tumours	N/A	Acetylation of p53 promotes the transcriptional activation of PUMA, leading to the death of KRAS‐mutant tumour cells.	N/A	^141^
	Liver cancer cells	K373, K382	During arsenic‐induced apoptosis, p53 acetylation and phosphorylation synergistically promote selective autophagic feedback‐mediated degradation of IKKα.	IKKα &	^142^
p300/CBP	Wilms tumour	K373, K382	Increasing CBP/p300‐mediated p53 acetylation can promote the expression of p53 target genes involved in the cell cycle, apoptosis and more.	WTX &	^143^
	Cervical cancer	K373	Knocking down CITED2 enhances p300/CBP‐mediated p53 acetylation, reducing p53 ubiquitination levels and protein accumulation and increasing cancer cell sensitivity to cisplatin treatment.	CITED2 #	^144^
CBP	N/A	N/A	Acetylated p53 activates p53‐mediated transcription.	lncRNA SCARNA10 &	^145^
Tip60	N/A	K120	Inhibiting TIP60 reduces p53 acetylation levels, thereby suppressing the activation of p21 and PUMA.	UHRF1 #	^146^
PCAF	5‐FU resistant colorectal cancer	N/A	Reduction of PCAF decreases p53 acetylation, weakening p53‐dependent transcriptional regulation of p21.	N/A	^147^
MOZ	Acute myeloid leukaemia	K120, K382	MOZ‐mediated p53 acetylation enhances p53‐dependent p21 transcription.	N/A	^148^
NAT10	Colon cancer	K120	NAT10 acetylates p53 to counteract MDM2 inhibition of p53, thereby stabilising p53.	N/A	^149^
PBRM1	Renal cancer	K382	PBRM1 uses its unique BD4 to recognise acetylation sites on p53 protein, promoting p21 transcription.	N/A	^150^

^a^ indicates upstream regulatory factors of p53 acetyltransferases; & indicates the positive regulatory relationship; # indicates the negative regulatory relationship; N/A indicates not available.

**TABLE 2:** Deacetylases of p53.

Deacetylase	Cancer type	Acetylation site	Function	Regulatory factor^a^	References
SIRT1	Hepatocellular carcinoma	K382	Dulcitol, an inhibitor of SIRT1, induces apoptosis by inhibiting the SIRT1/p53 pathway.	Dulcitol #	^151^
	N/A	N/A	Inhibiting SIRT1 stimulates p53 acetylation and promotes apoptosis in tumour cells.	TSPYL2 #	^135^
	Colon cancer	N/A	Deacetylation of p53 inhibits apoptosis in tumour cells.	N/A	^152^
	Breast cancer	N/A	Deacetylated p53 dissociates from the E3 ubiquitin ligase CBL, facilitating CBL recruitment of HIF‐1α for ubiquitination.	Vitamin C &	^153^
	Colon cancer, lung cancer, breast cancer	K370, K372, K373, K381, K382	Inhibition of SIRT1, which causes hyperacetylation of the C‐terminus of p53, can inhibit the formation of the p53/p21 complex and promote cell proliferation.	N/A	^154^
	Colorectal cancer	N/A	SIRT1‐mediated deacetylation of p53 inhibits cell apoptosis.	N/A	^155^
	Liver cancer cells	N/A	Inhibition of SIRT1 increases p53 acetylation levels, suppressing HBV replication and liver cancer cell proliferation.	Butyrate #	^156^
	Glioma	N/A	Down‐regulation of SIRT1 promotes the expression and acetylation of the p53 protein, inducing cell cycle arrest and apoptosis.	Sinomenine #	^157^
	Endometrial carcinoma	N/A	Inhibition of SIRT1 increases p53 acetylation levels, inducing p21‐mediated cell cycle arrest.	N/A	^158^
	Prostate cancer	N/A	Inhibition of SIRT1 reduces p53 deacetylation, enhancing doxorubicin‐induced mitochondrial apoptosis.	miR‐204 #	^159^
SIRT2	Non‐small cell lung cancer	N/A	Inhibiting SIRT2 leads to elevated p53 acetylation levels and increased expression of its transcriptional targets, such as the proapoptotic genes PUMA and NOXA.	N/A	^160^
SIRT7	Hepatocellular carcinoma	K320, K373	SIRT7‐mediated deacetylation of p53 decreases NOXA transcription, inhibiting apoptosis.	N/A	^161^
	N/A	K382	SIRT7‐mediated deacetylation of p53 hinders tumour progression by attenuating p53 activity.	N/A	^162^
HDAC1	Renal cell carcinoma	N/A	The deacetylated state of the p53 protein inhibits PGC1A transcriptional activity, leading to abnormal lipid accumulation within cells.	MIER2 &	^163^
	Epidermotropic T‐cell lymphoma	N/A	HDAC1‐mediated deacetylation of p53 leads to the suppression of downstream apoptosis‐related genes.	N/A	^164^
	Hepatocellular carcinoma	K382	HDAC1‐mediated deacetylation of p53 inhibits cell cycle arrest and apoptosis.	HOXA10 &	^165^
	N/A	K120	Inhibition of HDAC1 induces p53 acetylation, increasing the transcription of Apaf‐1, a key component of the mitochondrial apoptotic pathway, which mediates apoptosis.	N/A	^166^
	Ewing family tumours	K382	Activation of HDAC1 inhibits the deacetylation process of p53, weakening its transcriptional activity and promoting mdm2‐mediated degradation of p53.	EWS‐Fli1 &	^140^
HDAC2	Solid tumour	N/A	Activation of HDAC1 mediates p53 deacetylation, reducing p53 activity and promoting the proliferation of solid tumour cells.	TRB1 &	^167^
	Glioblastoma	N/A	HDAC2 maintains the self‐renewal of glioblastoma stem cells through p53 deacetylation.	TAGLN &	^168^
	N/A	N/A	Deubiquitylation of HDAC2 inhibits p53 acetylation, thereby weakening p53 transcriptional activity and DNA damage‐induced apoptosis.	UAP4 &	^169^
	Hepatocellular carcinoma	N/A	Silencing HDAC2 can increase p53 acetylation levels, inhibit cell proliferation and induce apoptosis.	N/A	^170^
	Gastrointestinal cancer	K320	Ubiquitination of HDAC2 at K462 leads to p53 deacetylation, thereby inhibiting the transcription of genes related to the cell cycle and apoptosis.	N/A	^171^
HDAC3	Melanoma	K373, K382	Inhibiting HDAC2 increases p53 acetylation and transcriptional activity.	Rg3 #	^172^
HDAC6	N/A	K381, K382	HDAC6 deacetylates p53 and stabilises Hsp90, thereby inhibiting p53‐induced apoptosis.	N/A	^173^
HDAC8	Acute myeloid leukaemia	N/A	HDAC8‐mediated deacetylation of p53 can promote the transformation and maintenance of leukaemia stem cells.	N/A	^174^

^a^ indicates upstream regulatory factors of p53 deacetylases; & indicates the positive regulatory relationship; # indicates the negative regulatory relationship; N/A indicates not available.

#### 3.4.3 Acetylation of SREBP

Sterol‐regulatory element binding proteins (SREBPs) function as intracellular detectors of cholesterol and are situated within the endoplasmic reticulum. When intracellular cholesterol is abundant, SREBP2 is anchored to the endoplasmic reticulum. When cholesterol levels decrease, SREBPs are cleaved and released, acting as transcription factors that bind to the gene operators of LDL receptors or HMG‐CoA synthase (HMGCS) to regulate cellular lipid metabolism.^175^ There are three main isoforms of activated SREBP: SREBP‐1a, SREBP‐1c and SREBP‐2.^175^ mTORC1 promotes lipid synthesis by activating the transcription factor SREBP1. SREBP1 is downstream of mTORC1 signalling and is a key target in the mTORC1‐mediated regulation of lipid synthesis.^176^ The omega‐3 polyunsaturated fatty acid docosahexaenoic acid induces the expression of SIRT1 in human colon epithelial cells. SIRT1 inhibits SREBP1 activity by deacetylating SREBP1, down‐regulates COX‐2 expression and consequently reduces the impact of obesity‐related inflammation on colon cancer.^177^ SREBP‐1c primarily regulates the expression of genes controlling fatty acid synthesis. SREBP‐1c is acetylated and deacetylated by p300 and SIRT1 at lysine 289 and lysine 309, respectively. p300 promotes the transcriptional activity of SREBP‐1c through acetylation, stabilises SREBP‐1c and increases the expression of fatty acid synthesis genes.^178^

#### 3.4.4 Acetylation of HIF1α

Hypoxia induces the expression of the HIF‐1α protein, which is an α subunit of hypoxia‐inducible factor (HIF‐1). HIF‐1 is a transcription factor formed by the combination of two subunits, namely HIF1α and HIF1β, resulting in a heterodimeric structure. As solid tumours gradually expand during growth, host blood vessels cannot meet their growth demands, resulting in a hypoxic environment.^179^ Under hypoxia or stimulation by certain cytokines, the expression of the HIF1α subunit significantly increases, and HIF1α enters the nucleus to bind to the HIF1β subunit to form active HIF‐1, which then regulates the transcription of downstream target genes, promoting the expression of glycolysis‐related enzymes and allowing tumour cells to produce energy through glycolysis even under hypoxic conditions,^180^ while mTORC1 can induce HIF1α expression to increase the expression of glucose transporters and glycolytic genes.^181^, ^182^ PCAF acetylates lysine 674 of HIF1α, and while SIRT1 deacetylates this site, deacetylation inactivates HIF1α, inhibits HIF1α target genes and blocks glycolysis and redox responses.^183^ Therefore, SRT1720, a SIRT1 activator, can significantly inhibit the growth of bladder cancer cells and may become a new method for treating bladder cancer.^184^ Under chronic hypoxic conditions, NAD+ levels decrease, leading to SIRT1 inactivation. Lysine 709 of HIF1α is acetylated by p300, and acetylated HIF1α is recognised by VHL, resulting in ubiquitin/proteasome degradation.^185^

In pancreatic cancer, MTA2 stabilises and activates HIF1α through deacetylation, and the activated HIF1α subsequently regulates the transcription of the MTA2 transcriptional regulator LncRNA–MTA2TR, thereby affecting the activity of MTA2.^186^ HIF1α can also promote the transcription of miR‐646 in pancreatic cancer, and MIIP, a target gene of miR‐646, can inhibit the acetylation effect of HDAC6 on HIF1α in deacetylation, promoting the degradation of HIF1α.^187^ The accumulation of HDAC4 in the nucleus reduces the acetylation level of HIF1α, enhancing the stability and transcriptional activity of HIF1α and increasing the adaptability of tumour cells to hypoxic environments.^188^, ^189^, ^190^ HAUSP (USP7), a deubiquitinating enzyme, can both deubiquitinate HIF‐1α to increase its stability and interact with CBP to mediate the acetylation of H3K56, enhancing the transcription of HIF‐1α and thereby inducing tumour EMT.^191^ In breast cancer, HEXIM1 can weaken the interaction between HDAC1 and HIF‐1α, leading to the acetylation of HIF‐1α and reducing the expression of its target genes, thereby inhibiting cell invasion.^192^ MTA1 can promote the interaction between HDAC1 and HIF‐1α, inducing the deacetylation of HIF‐1α.^193^, ^194^ In HCC, PROX1 can recruit HDAC1 to deacetylate HIF‐1α, stabilising HIF‐1α and inducing EMT in cells.^195^ In lung cancer cells, connective tissue growth factor increases the expression of ARD‐1, which is also known as Naa10, an N‐terminal acetyltransferase that mediates the acetylation of HIF‐1α at Lys532, leading to its interaction with pVHL and subsequent degradation, thereby inhibiting tumour cell progression.^196^

#### 3.4.5 Acetylation of NFR2

Nuclear factor erythroid‐2‐related factor‐2 (NRF2), a key transcription factor that protects against oxidative stress, activates the expression of a range of antioxidant genes to help cells cope with oxidative stress.^197^ NRF2 may indirectly affect the stability or activity of HIF1α by influencing the intracellular redox state, for example, when cells are under the combined stress of oxidative stress and hypoxia, the activation of NRF2 may help cells scavenge ROS, thus mitigating the potentially negative effect on HIF1α stability.^198^ The role of NRF2 in cancer is bidirectional, as it can act as an oncogene to regulate intracellular oxidative stress and inflammatory responses and promote the growth and metastasis of tumour cells through intracellular metabolic pathways and signalling pathways.^199^ In HCC, SIRT1 deacetylates NRF2, leading to increased expression of key iron death‐related genes, proteins and molecules such as SLC7A11, GPX4 and GSH under NRF2 transcriptional activation. This elevation subsequently inhibits ferroptosis, enhancing cell survival and colony formation ability in liver cancer cells.^200^ ARF, a tumour suppressor gene, plays a crucial role in activating p53 during oncogenic stress. ARF can inhibit the CBP‐mediated acetylation of NRF2, thereby reducing NRF2 transcriptional activity rather than NRF2 stability, down‐regulating SLC7A11 expression and increasing the likelihood of iron death in p53‐independent cells.^201^ Therefore, the acetylation modification of NRF2 can either enhance or inhibit its transcriptional activity, with different acetylation sites potentially playing a decisive role. The histone acetyltransferase MOF is a member of the MYST family. In human non‐small cell lung cancer, hMOF expression is up‐regulated. hMOF directly acetylates NRF2 at K588, enhancing NRF2 nuclear retention and transcription of downstream genes. This process further promotes tumour growth and resistance to chemotherapy drugs.^202^ NRF2 overexpression is considered to be the driving factor in the tumour progression stage. In colon cancer, ARD‐1 promotes tumour progression by directly acetylating NRF2 and enhancing its stability.^203^

#### 3.4.6 Acetylation of c‐myc

c‐myc is a proto‐oncogene, and the protein encoded by this gene is a key regulator of cell growth and proliferation, affecting various biological processes, such as the cell cycle, apoptosis, cell differentiation and metabolism.^204^ As a transcription factor, c‐myc can activate glycolytic genes and glucose transporters, ultimately enhancing the glycolytic capacity of cells to meet the energy demands of rapidly proliferating tumour cells. c‐myc promotes the production and export of lactate by increasing the gene expression of LDHA and the lactate transporter MCT1, helping to maintain the acidic microenvironment of tumour cells and thus promoting tumour invasion and metastasis.^205^ In HCC, KAT5 can enhance the expression of MMP9 and MMP14 through the acetylation of c‐myc, thereby promoting EMT. Phosphoenolpyruvate carboxykinase 1 can mediate the ubiquitination of KAT5 through O‐β‐acetylglucosamine modification.^206^ In undifferentiated thyroid carcinoma, KAT5 can also stabilise c‐myc through acetylation, promoting tumour invasion and metastasis.^207^ In non‐small cell lung cancer, circRHOT1 recruits KAT5 to promote H3K27 acetylation, increasing RNA polymerase II enrichment at the c‐myc promoter and c‐myc expression.^208^ ASF1B is highly expressed in pancreatic adenocarcinoma patient samples. Mechanistically, ASF1B increases H3K56 histone acetylation in the c‐myc promoter region in a CBP‐dependent manner, activating c‐myc transcription and thereby promoting pancreatic cancer progression.^209^ Hyperlipidaemia can impair the tumour cell immune response, and in a high‐fat environment, high expression of c‐myc can enhance the anti‐tumour efficacy of NK cells. In vitro exposure to oleic acid reduces p300‐mediated c‐myc acetylation, shortens the half‐life of the c‐myc protein and reduces p300‐mediated H3K27 histone acetylation, ultimately leading to persistent natural killer cell dysfunction.^210^ In multiple myeloma and HCC, the ubiquitin ligases SIAH2 and TRAF6 can degrade HDAC3 through ubiquitination, enhancing H3K27 and H3K9 acetylation levels in the c‐myc promoter region and thereby promoting c‐myc transcription.^211^, ^212^ In renal cell carcinoma, the HDAC3 protein can also inhibit c‐myc transcription by reducing histone H3 deacetylation.^213^ However, in CCA, HDAC3 plays a carcinogenic role by directly deacetylating the c‐myc K323 site, stabilising the c‐myc protein, reducing pyruvate levels, and thereby promoting CCA cell proliferation.^214^ In colorectal cancer, the lncRNA NEAT1 can also influence chromatin remodelling by increasing H3K27 acetylation levels, promoting c‐myc transcription and affecting patient responses to 5‐FU treatment.^215^ Lysyl‐oxidase like‐2 (LOXL2) can also interact with H3 histones, deacetylate H3K36 and block the transcription of H3K36 acetylation‐dependent genes, such as c‐myc and HIF1α, thereby inhibiting the growth of transplanted tumours in vivo.^216^ In renal cell carcinoma, the pVHL protein, in addition to regulating HIF1α expression, can inhibit c‐myc transcription by enhancing HDAC1/2‐mediated histone deacetylation of the c‐myc promoter.^217^ In cervical cancer, GCN5 increases the binding of GCN5 to the E2F1 promoter region by acetylating c‐myc and increases the degree of histone acetylation in this region, accelerating E2F1 expression and cell cycle progression.^218^ In leukaemia cell lines, SIRT1 can also directly interact with c‐myc, causing its deacetylation. A decrease in the acetylation level of c‐myc promotes the transcriptional activity of c‐myc and accelerates cell proliferation.^219^

### 3.5 Acetylation and metabolic enzymes

#### 3.5.1 Acetylation and glycolysis

**FIGURE 4:**
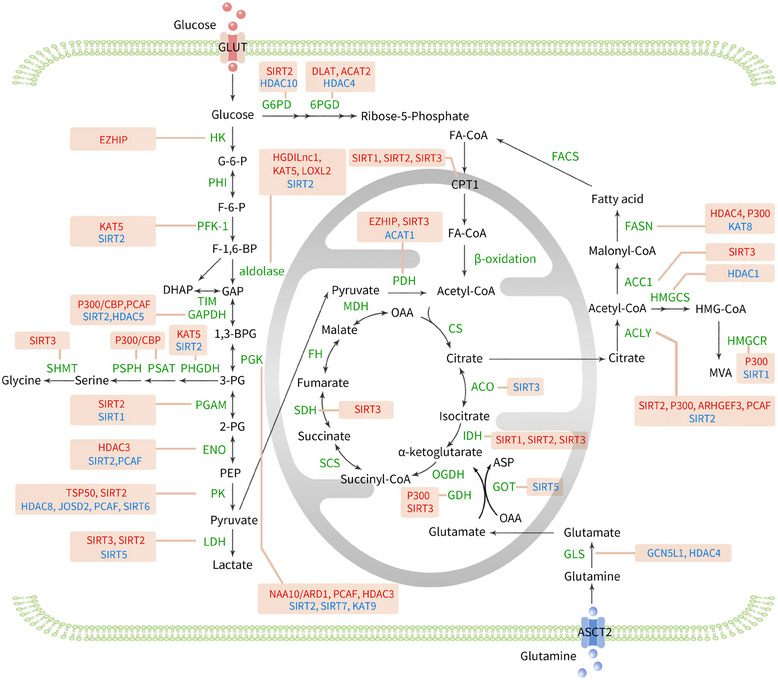
In the regulation of cancer metabolism, metabolic enzymes involved in glucose, fatty acid and amino acid metabolic pathways are finely regulated by acetylation and deacetylation processes, thereby achieving tight control of cancer cell metabolic pathways. Red text indicates the positive regulatory relationship of acetyltransferases and deacetylase on protein activity or expression levels. Blue text indicates the negative regulatory relationship of acetyltransferases and deacetylase on protein activity or expression levels. 1,3‐BPG, 1,3‐biphosphoglycerate; 2‐PG, 2‐phosphoglycerate; 3‐PG, 3‐phosphoglycerate; 6PGD, 6‐phosphogluconate dehydrogenase; ACAT, acetyl‐CoA acetyltransferase; ACC, acetyl‐CoA carboxylase; ACLY, ATP citrate lyase; ACO, cis‐aconitase; ASCT2, alanine‐serine‐cystenine transporter2; Asp, aspartate; CPT, carnitine palmitoyltransferase; CS, citrate synthase; DHAP, dihydroxyacetone phosphate; DLAT, dihydrolipoamide S‐acetyltransferase; ENO, enolase; EZHIP, enhancer of zeste homologs inhibitory protein; F‐1,6‐BP, fructose‐1,6‐bisphosphate; F‐6‐P, fructose‐6‐phosphate; FA‐CoA, fatty acyl‐CoA; FACS, fatty acyl‐CoA synthetase; FASN, fatty acid synthase; FH, fumarase; G‐6‐P, glucose‐6‐phosphate; G6PD, glucose 6‐phosphate dehydrogenase; GAP, glyceraldehyde‐3‐phosphate; GAPDH, glyceraldehyde‐3‐phosphate dehydrogenase; GDH, glutamate dehydrogenase; GLS, glutaminase; GLUT, glucose transporter; GOT, glutamate: oxaloacetate transaminase; HGDILnc1, hypoxia and glucose deprivation‐induced lncRNA; HK, hexokinase; HMGCR, HMG‐CoA reductase; HMGCS, HMG‐CoA synthase; IDH, isocitrate dehydrogenase; JOSD2, Josephin domain containing 2; LDH, lactate dehydrogenase; LOXL2, lysyl oxidase‐like 2; MD, malate dehydrogenase; OAA, oxaloacetate; OGDH, α‐ketoglutarate dehydrogenase; PDH, pyruvate dehydrogenase; PEP, phosphoenolpyruvate; PFK‐1, phosphofructokinase‐1; PGAM, phosphoglycerate mutase; PGK, phosphoglycerate kinase; PHGDH, 3‐phosphoglycerate dehydrogenase; PHI, phosphohexose isomerase; PK, pyruvate kinase; PSAT, phosphoserine aminotransferase; PSPH, phosphoserine phosphatase; SCS, succinyl‐CoA synthetase; SDH, succinate dehydrogenase; SHMT, serine transhydroxymethylase; TIM, triose phosphate isomerase; TSP50, testes‐specific protease 50.

Posterior fossa group A ependymomas (PFA) are a type of paediatric central nervous system tumour with poor conventional treatment outcomes and prognosis. EZHIP (enhancer of Zeste homologs inhibitory protein) is up‐regulated in PFA. EZHIP can increase the expression of these enzymes by enhancing histone acetylation, thereby promoting glycolysis and tricarboxylic acid cycle metabolism and advancing lethal PFAs in children^220^ (Figure 4).

SIRT2 inhibits phosphofructokinase‐platelet type (PFKP) by deacetylating lysine 395, the second rate‐limiting enzyme of glycolysis, thus inhibiting glycolysis.^221^ EGFR activation leads to KAT5‐mediated acetylation of lysine 395 of PFKP, causing its translocation to the cell membrane. Subsequently, the PI3K/AKT signalling pathway is activated, enhancing the phosphorylation of PFK2, thereby activating PFK1 and increasing GLUT1 expression, thus promoting glycolysis and tumour cell proliferation.^222^

The aldolase enzyme family (ALDO) represents the fourth enzyme participating in the glycolytic pathway. This family can be categorised into three distinct isoforms, ALDOA, ALDOB and ALDOC, on the basis of their expression in different human organs.^223^ Hypoxia and low‐glucose environments can enhance angiogenesis. At the mechanistic level, hypoxia and glucose deprivation‐induced lncRNA (HGDILnc1) has the capacity to increase the acetylation of histone H2B at lysine 16 within the promoter region of ALDOC. This modification facilitates the transcription of ALDOC, ultimately leading to increased glycolysis and angiogenesis.^224^ MiR‐200c‐5p can also target and silence SIRT2, thereby increasing the acetylation levels of various glycolytic enzymes, which enhances their activities, such as aldolase, glyceraldehyde‐3‐phosphate dehydrogenase (GAPDH), phosphoglycerate kinase and enolase, thus enhancing the molecular characteristics of pluripotent stem cells.^225^ ZNF692 is a transcription factor belonging to the Krüppel‐like zinc finger protein family and a key cellular metabolic sensor.^226^ ZNF692 promotes KAT5 transcription and increases the acetylation level of ALDOA, thereby accelerating glycolysis and promoting the progression of HCC.^227^ LOXL2 and its catalytically inactive isoenzyme L2Δ13 have been shown to be new deacetylases that can directly deacetylate lysine 13 of ALDOA, enhancing glycolysis and promoting metabolic reprogramming and the progression of oesophageal cancer.^228^

GAPDH oxidises glyceraldehyde‐3‐phosphate, which is the sole oxidation‒reduction reaction in the entire glycolytic pathway. In the Neuro2a neuroblastoma cell line, homocysteine induces p300/CBP‐mediated nuclear translocation and acetylation of GAPDH,^229^ thereby enhancing the catalytic and acetylation activities of p300/CBP, activating downstream molecules such as p53 and promoting apoptosis.^230^ It has also been reported that GAPDH nuclear translocation may be mediated by acetylation of three specific lysine residues (117, 227 and 251).^231^ Under glucose stimulation, PCAF can acetylate lysine 254 of GAPDH, increasing GAPDH activity, while HDAC5 can deacetylate the corresponding site, reversing GAPDH activity, thereby regulating cellular metabolism and altering tumour cell growth.^232^

Under glutamate deprivation and hypoxic conditions, phosphoglycerate kinase 1 (PGK1) is acetylated at lysine 388 by NAA10/ARD1, which subsequently leads to the interaction of PGK1 with Beclin 1, causing phosphorylation of Beclin 1 at serine 30, thereby inducing autophagy and brain tumour formation.^233^, ^234^ PCAF and SIRT7 bidirectionally regulate the acetylation level of PGK1 at lysine 323 in HCC cells, promoting or inhibiting the enzymatic activity of PGK1, thereby altering glycolysis processes and controlling the proliferation of HCC cells.^235^ The acetylation of PGK1 at lysine 220 impedes its enzymatic function by interfering with its interaction with the substrate ADP. KAT9 and HDAC3 may serve as potential acetyltransferases and deacetylases for PGK1 K220. The phosphorylation of HDAC3 at serine 424 is modulated by the PI3K/AKT/mTOR signalling pathway, promoting HDAC3's deacetylation activity and thereby governing glycolytic ATP synthesis and maintaining the cellular redox balance.^236^

Phosphoglycerate mutase 5 (PGAM5) is a member of the phosphoglycerate mutase family. SIRT2 mediates PGAM5 deacetylation, activating PGAM5, which subsequently mediates the phosphorylation of malate dehydrogenase to activate malic enzyme 1, promoting NADPH production and indirectly facilitating lipid synthesis and HCC cell proliferation.^237^ PGAM K100 is an active site residue that is highly conserved across different species. K100 acetylation reduces PGAM2 activity, while SIRT2 deacetylates and activates PGAM2, increasing NADPH production and promoting tumour cell growth.^238^ Under glucose restriction conditions, SIRT1 levels significantly increase, which can also lead to PGAM1 deacetylation and reduced activity, inhibiting the process of glucose‐to‐fat burning.^239^

Enolase catalyses the conversion of 2‐phosphoglycerate to phosphoenolpyruvate. In pancreatic ductal adenocarcinoma (PDAC), enolase 2 (ENO2) expression is significantly increased. K394 is the main acetylation site at which acetylation regulates ENO2 enzymatic activity. HDAC3 and PCAF are potential deacetylases and acetyltransferases for ENO2, respectively. HDAC3‐mediated deacetylation of ENO2 K394 activates ENO2, enhancing glycolysis. Insulin‐like growth factor‐1 promotes HDAC3 phosphorylation at serine 424 in a dose‐ and time‐dependent manner through the PI3K/AKT/mTOR pathway, activating HDAC3, inducing ENO2 deacetylation, negatively regulating glycolysis and preventing PDAC development.^240^ Recurrent chromosomal translocations are characteristic of many human cancers. TFG‐TEC is a chimeric protein formed by the fusion of two genes, and its oncogenic effect may be due to its increased transcriptional activation capacity. Studies have shown that TFG‐TEC activates the transcription of endogenous β‐enolase by promoting the expression of the histone H3 acetylation, triggering the expression of metabolism‐related genes in cancer cells and promoting the progression of human extra skeletal myxoid chondrosarcomas.^241^

The M2 isoform of pyruvate kinase (PKM2) is the third rate‐limiting enzyme in glycolysis and plays an important role in cell metabolism and proliferation. HDAC8 interacts with and removes acetyl groups from the K62 residue of PKM2, promoting its nuclear entry and binding to β‐catenin, thus facilitating the transcription of the CCND1 gene and advancing the progression of the cell cycle.^242^ The deubiquitinase JOSD2 is a novel tumour suppressor, and research has revealed that PKM2 is a new protein that interacts with JOSD2. JOSD2 inhibits nuclear localisation by reducing the lysine 433 acetylation of PKM2 in acute myeloid leukaemia (AML), thereby decreasing downstream gene expression and ultimately slowing AML progression.^243^ Testes‐specific protease 50 (TSP50) has been identified as an oncogene. It can also bind to PKM2, promoting the Warburg effect and proliferation of HCC cells by increasing acetylation at PKM K433.^244^ PCAF can also acetylate PKM2 at K305, leading to its lysosomal degradation via chaperone‐mediated autophagy.^245^ SIRT2‐deficient breast cancer cells exhibit increased acetylation at PKM2 K305, preventing PKM2 tetramerisation and thereby reducing PKM2 enzymatic activity, ultimately altering glucose metabolism and inhibiting tumour growth.^246^ SIRT6 binds and deacetylates nuclear PKM2 at K433, leading to the expulsion of PKM2 from the nucleus, thus eliminating its role as a nuclear protein kinase and a transcriptional coactivator, and resulting in decreased tumour cell proliferation, migration and invasion.^247^

LDH is a glycolytic enzyme that catalyses the conversion of pyruvate and NADH to lactate and NAD (+), which is crucial for maintaining the balance of lactate and pyruvate, energy production and regulation of the cellular redox state.^248^ LDHB is a subunit of LDH. SIRT5 can bind to LDHB and promote LDHB enzyme activity by deacetylating lysine 329 on LDHB, thereby enhancing autophagy and accelerating the growth of colon cancer cells.^249^ SIRT3 can interact with another subunit of LDHA and deacetylate it, enhancing LDHA activity and promoting glycolysis and gastric cancer cell proliferation.^250^ In pancreatic cancer tissues, SIRT2 deacetylates LDHA at the K5 site, preventing its recognition by the HSC70 chaperone and inhibiting its lysosomal degradation, thereby increasing LDHA activity and protein levels, enhancing the Warburg effect and promoting pancreatic cancer cell proliferation and migration.^251^

### 3.6 Acetylation and the tricarboxylic acid cycle

Pyruvate dehydrogenase (PDH) converts pyruvate into acetyl‐CoA through decarboxylation, which is then utilised by the TCA cycle and oxidative phosphorylation in normal and cancer cells to produce ATP. Therefore, PDH links glycolysis to the TCA cycle. Acetyl‐CoA acetyltransferase 1 (ACAT1) generates acetoacetyl‐CoA from acetyl‐CoA in liver mitochondria, which is used for ketone body formation. Studies have shown that ACAT1 also has acetyltransferase activity, specifically acetylating PDH, reducing its enzymatic activity, increasing pyruvate levels and promoting lactate production and tumour growth through the Warburg effect, making ACAT1 a potential new anti‐cancer target.^252^, ^253^ SIRT3 is the main mitochondrial deacetylase and can directly target PDH to maintain its optimal catalytic activity, enhancing pyruvate oxidation and inhibiting the Warburg effect and lactate production.^253^, ^254^, ^255^ In human prostate tumours, the androgen receptor binds to its coregulator, steroid receptor coactivator‐2, to recruit HDAC2 to the SIRT3 promoter, inhibiting SIRT3 transcription, subsequently increasing the level of the aconitase acetylation, significantly enhancing ACO2 activity, and promoting the progression of advanced prostate cancer.^256^

Isocitrate dehydrogenase (IDH) facilitates the oxidative decarboxylation process of isocitrate, resulting in the formation of α‐ketoglutarate, a key step in the TCA cycle. Lycorine, a flavonoid compound with various biological activities, can directly target the unique C‐terminal domain of IDH1, disrupting the interaction between IDH1 and SIRT1, thereby promoting IDH1 acetylation, inhibiting IDH1 enzymatic activity and inducing oxidative stress, mitochondrial membrane damage and mitochondrial fission in CRC cells.^257^ TNFα can also disrupt the interaction between SIRT1 and IDH1, increasing the acetylation level of IDH1 at K115, leading to IDH1 ubiquitination and enhancing the sensitivity of CRC cells to 5‐FU. Therefore, the high acetylation of IDH1 at K115 induced by TNFα can serve as a marker for predicting the response of CRC patients to chemotherapy.^258^ SIRT2 deacetylates IDH1 at K224, promoting its enzymatic activity and α‐ketoglutarate production and inhibiting the invasion and migration of CRC cells, while high acetylation of IDH1 at K224 is significantly associated with distant metastasis and survival in colorectal cancer patients.^259^ SIRT3 deacetylates IDH2 at K413, which activates its enzymatic activity by promoting IDH2 dimer formation, subsequently reducing intracellular ROS and glycolysis, and inhibiting the tumour‐prone phenotype.^260^, ^261^, ^262^ c‐myc promotes the degradation of SIRT3 deacetylase mediated by the cell cycle regulatory protein SKP2, leading to acetylation‐dependent inactivation of succinate dehydrogenase complex subunit A at K335, resulting in succinate accumulation in cells and triggering H3K4me3 activation and tumour‐specific gene expression, thereby promoting tumour growth.^263^

### 3.7 Acetylation and the pentose phosphate pathway

The pentose phosphate pathway is another crucial metabolic pathway that generates NADPH and ribose‐5‐phosphate, which are involved in nucleotide biosynthesis and redox balance. Glucose‐6‐phosphate dehydrogenase (G6PD) is the first rate‐limiting enzyme of this pathway, and SIRT2 is involved in regulating the acetylation of G6PD. In lung adenocarcinoma cells, Krüppel‐like factor 8 activates SIRT2 transcription.^264^ In HCC, TSP50 promotes the binding of G6PD to SIRT2.^265^ Subsequently, deacetylation decreases G6PD ubiquitination and enhances SUMO1 modification, increasing G6PD stability, promoting tumour cell proliferation and inhibiting apoptosis.^266^ In AML, SIRT2 deacetylates G6PD at the K403 site, forming an active dimer,^267^ which promotes NADPH production, thereby enhancing leukaemia cell proliferation and clonogenic activity.^268^ In addition to SIRT2, HDAC10 can also reduce G6PD transcription by inhibiting the expression of the histone acetylation, decreasing lung cancer cell proliferation and tumour growth. 4‐Hydroxyphenylpyruvate dioxygenase (HPD) serves a crucial role in the regulation of tyrosine metabolism. It facilitates the breakdown of tyrosine, which results in elevated levels of acetyl‐CoA. Moreover, HPD is involved in the translocation of HDAC10 from the nucleus to the cytoplasmic compartment via LKB1–AMPK signalling, both of which enhance histone acetylation, boosting G6PD transcription.^269^

6‐Phosphogluconate dehydrogenase (6PGD) is another key rate‐limiting enzyme in the pentose phosphate pathway. In human cancer cells, the acetylation of 6PGD at the K76 and K294 sites promotes the binding of NADP (+) with 6PGD and the formation of active 6PGD dimers, thereby increasing the production of ribose‐5‐phosphate and NADPH, which favour cancer cell growth and division. DLAT and ACAT2 are upstream acetyltransferases, while HDAC4 is the deacetylase for these enzymes. The expression of non‐acetylated 6PGD mutants in cancer cells markedly diminishes both cellular proliferation and tumour development.^270^

### 3.8 Acetylation and fatty acid oxidation

Carnitine palmitoyl transferase 1 (CPT1) transfers long‐chain acyl‐CoA from the cytosol to the mitochondrial matrix and is the rate‐limiting enzyme in the fatty acid oxidation (FAO) process. CPT1A is an isoform of CPT1. SIRT1 deacetylates CPT1A at the K675 site, inhibiting its ubiquitination and degradation and thereby promoting FAO. Berberine can partially alleviate non‐alcoholic fatty liver disease in mice by promoting SIRT1‐mediated deacetylation of CPT1A.^271^ Ketohexokinase‐C (KHK‐C) is the first‐step enzyme that catalyses fructose breakdown. Increased KHK‐C is associated with CPT1A acetylation at the K508 site and reduced CPT1A protein levels, leading to increased triglyceride accumulation. KHK‐C mainly regulates CPT1A‐mediated FAO by increasing the protein acetylation through decreasing SIRT2.^272^ Additionally, decreased SIRT3 activity leads to increased acetylation of CPT2 at K79, blocking FAO and exacerbating platelet storage lesions. Thus, CPT2 K79 acetylation might be a new target for improving platelet storage quality.^273^ Enoyl‐CoA hydratase‐1 (ECHS1) is an enzyme involved in FAO. Excess nutrients promote ECHS1 K101 acetylation, subsequently inducing ECHS1 ubiquitination and preventing its mitochondrial translocation, thereby inhibiting ECHS1 activity and leading to fatty acid accumulation and oncogenic mTOR activation. However, the oncogenic effects induced by nutrient excess can be reversed by SIRT3.^274^

### 3.9 Acetylation and fatty acid synthesis

ATP citrate lyase (ACLY) catalyses the conversion of citrate and coenzyme A to acetyl‐CoA and oxaloacetate, acting as a bridge between carbohydrate metabolism and lipid metabolism. In oesophageal squamous cell carcinoma, the ACLY protein carries acetylation modifications. SIRT2 interacts with ACLY, reducing the acetylation level of the ACLY protein, promoting ACLY activity and increasing lipid synthesis as well as cell proliferation, migration and invasion.^275^ The micro peptide ACLY‐BP encoded by LINC000887, which is inhibited by GATA3, maintains ACLY acetylation, preventing ACLY ubiquitination and degradation and stabilising ACLY to produce acetyl‐CoA, resulting in lipid synthesis and promoting cell proliferation in renal clear cell carcinoma.^276^ Branched‐chain amino acid transaminase 2 (BCAT2) is an oncogene in melanoma. BCAT2 attenuates P300‐dependent histone acetylation at the ACLY promoter, thereby inhibiting ACLY transcription. ZEB1 is an upstream transcription factor of BCAT2.^277^ ARHGEF3, a member of the Rho GEF family, is highly expressed in non‐small cell lung cancer. ARHGEF3 enhances ACLY protein stability by reducing acetylation at Lys17 and Lys86 of ACLY, leading to the dissociation of ACLY from its E3 ligase, NEDD4, making it a promising therapeutic target in non‐small cell lung cancer.^278^ In lung cancer, the ACLY lysine residues at positions 540, 546 and 554 can also be acetylated. High glucose stimulates the acetylation of these three lysine residues via PCAF, increasing ACLY stability by blocking its ubiquitination and degradation and promoting de novo lipid synthesis, cell proliferation and tumour growth. SIRT2 can deacetylate these residues and destabilise them.^279^ The same deacetylase can have different regulatory effects on ACLY in tumours at different sites, so the effects of acetylation on tumours cannot be separated from the heterogeneity of organs.

Acetyl‐CoA carboxylase (ACC1) can catalyse the synthesis of malonyl‐CoA from acetyl‐CoA and is the rate‐limiting enzyme in fatty acid synthesis reactions; its activity directly affects the rate of fatty acid synthesis. SIRT3 up‐regulates ACC1 expression by deacetylating ACC1, significantly enhancing fatty acid synthesis reprogramming in cervical squamous cell carcinoma, thereby promoting cell proliferation and metastasis.^280^

Fatty acid synthase (FASN) is the terminal enzyme in fatty acid synthesis and plays a key role in cell proliferation. KAT8 and HDAC4 act as the acetyltransferase and deacetylase of FASN, respectively, regulating FASN protein levels and thereby altering fatty acid synthesis and cell growth in liver cancer cells. The acetylation of FASN enhances its binding to the E3 ubiquitin ligase TRIM21, promoting its degradation through the ubiquitin‒proteasome pathway and thereby reducing its stability.^281^ Studies related to prostate cancer have shown that p300 promotes tumour growth and regulates the expression of the lipid metabolism regulator FASN by acetylating H3 in the FASN gene promoter. Thus, p300 may be involved in the regulation of lipid metabolism.^282^ BCAT2 promotes melanoma progression by epigenetically regulating FASN expression through the P300‐dependent histone acetylation.^277^ In the cytoplasm, in addition to citrate being catalysed by ACLY to produce acetyl‐CoA, acetate can also generate acetyl‐CoA under the catalysis of acetyl‐CoA synthetase to increase protein acetylation levels. Studies have shown that acetate activates FASN gene transcription by increasing the acetylation levels of H3K9, H3K27 and H3K56 in the FASN gene promoter region, thereby enhancing de novo lipid synthesis and tumour growth. Acetyl‐CoA synthetase is involved in this acetate‐mediated epigenetic regulation.^283^

### 3.10 Acetylation and cholesterol synthesis

HMGCS mainly participates in the process of cholesterol synthesis in organisms and plays an important role in the early stages of cholesterol synthesis. In colon cancer cell lines, HDAC1 is associated with the endogenous mitochondrial HMGCS promoter. Overexpression of HDAC1 leads to hypoacetylation of the mitochondrial HMGCS promoter and reduces its transcriptional activity. The specific HDAC inhibitor trichostatin A can induce transcriptional activity and mRNA expression of this gene.^284^ HMG‐CoA reductase (HMGCR) plays an important role in regulating blood lipids. It catalyses the substrate HMG‐CoA to produce mevalonate, which is the rate‐limiting step in the synthesis of cholesterol in the body. In mammalian cells, P300 can acetylate the H3K27 site of the HMGCR promoter. 25‐Hydroxycholesterol inhibits the recruitment of p300 to the HMGCR promoter and suppresses H3K27 acetylation and gene transcription.^285^ SIRT1 can mediate the deacetylation of H3K9ac and H3K14ac histones in the HMGCR promoter region, thereby reducing HMGCR transcription and expression. Clinical and animal researches have indicated that hypercholesterolemia and associated disorders have developmental origins that can be traced back to the intrauterine environment. High levels of glucocorticoids in utero activate the CCAAT enhancer‐binding protein α signalling pathway in the foetal liver and down‐regulate SIRT1 expression, thereby increasing HMGCR expression levels and promoting cholesterol synthesis. This glucocorticoid‐dependent cholesterol metabolic programming effect persists into adulthood, leading to the occurrence of hypercholesterolemia.^286^ However, the regulatory role of HMGCR acetylation levels in tumour cells has not been reported.

### 3.11 Acetylation and amino acid metabolism

Extracellular glutamine is transported into the cell via the amino acid transporter ASCT2 and hydrolysed to glutamate by glutaminase. Glutamate undergoes conversion to α‐ketoglutarate through the action of the enzyme glutamate dehydrogenase (GDH) or transaminases (ASTs/GOTs), which then enter the TCA cycle. Additionally, glutamine and the newly produced aspartate can be used for the de novo synthesis of purine nucleotides. In liver cancer, GCN5L1 acetylates glutaminase, inhibiting its activity and the mTORC1 pathway, thereby controlling the development of liver cancer.^287^ HDAC4 deacetylates glutaminase at lysine 311, inhibits K63‐linked ubiquitination of glutaminase, enhances enzyme activity and thus promotes tumourigenesis in non‐small cell lung cancer.^288^ Therefore, interfering with tumour progression by acetylating glutaminase is a promising cancer metabolism inhibition strategy.

In colon cancer cells, hypoxia induces p300 to recruit GDH1, promoting its acetylation at K503 and K527. Acetylation of GDH1 at K527 induces the formation of a GDH1 complex with EGLN1/HIF‐1α, whereas acetylation at K503 enhances its ability to react with α‐ketoglutarate to produce glutamate, increasing the stability of HIF‐1α and promoting the progression of CRC.^289^ In diffuse large B‐cell lymphoma, SIRT3 depletion reduces GDH deacetylation, decreases GDH stability, reduces glutamate flux into the tricarboxylic acid cycle and diminishes the acetyl‐CoA pool, thereby inducing autophagy and cell death.^290^ SIRT5 plays a role in suppressing tumour cell proliferation and is linked to a positive prognosis in individuals diagnosed with PDAC. Loss of SIRT5 enhances glutamate metabolism through acetylation‐mediated activation of transaminases, promoting the progression of KRAS‐induced PDAC.^291^

Phosphoglycerate dehydrogenase (PHGDH) is the first enzyme in the serine synthesis pathway and the rate‐limiting enzyme, helping 3‐phosphoglycerate leave glycolysis to generate serine. In breast cancer tissues, PHGDH expression is significantly up‐regulated. Tip60 (KAT5) and SIRT2 regulate PHGDH protein expression by reversibly modulating its acetylation. Acetylation of PHGDH at the K58 site disrupts the interaction between the E3 ubiquitin ligase RNF5 and PHGDH, reducing PHGDH degradation and increasing enzyme stability, thereby promoting breast cancer cell proliferation.^292^ The novel p300 inhibitor B029‐2 targets p300/CBP, reducing the expression of serine synthesis enzymes (such as PSPH and PSAT1) by lowering H3K18Ac and H3K27Ac levels in gene promoter regions, thereby decreasing amino acid synthesis in liver cancer cells and resulting in significant anti‐tumour effects.^293^ Serine hydroxymethyltransferase 2 (SHMT2) converts serine to glycine, and the resulting glycine can serve as a substrate for de novo purine nucleotide synthesis. SIRT3 enhances the activity of the tetrameric structure of SHMT2 by deacetylating it at Lys 95, inhibiting its degradation by the K63‐ubiquitin proteasome and increasing the level of the reducing coenzyme NADPH, thereby promoting colorectal cancer cell proliferation.^294^

## 4 DISCUSSION

In the in‐depth exploration of tumour biology, the intricate molecular mechanisms involved in tumour biology and metabolic reprogramming are receiving increasing attention. This reprogramming is an adaptive strategy adopted by tumour cells to cope with rapid proliferation and survival pressure and involves changes in multiple metabolic pathways. Acetylation, an important form of epigenetic regulation, plays a crucial role in the fine‐tuning of tumour metabolism. Notably, tumour metabolic stress, such as nutrient deficiency, hypoxia or oxidative stress, can significantly affect the acetylation and deacetylation status within cells. This stress may further promote tumour cell adaptability and invasiveness by altering the epigenetic landscape. Conversely, changes in acetylation and deacetylation status can also influence the metabolic patterns of tumour cells, leading to the formation of an interactive dynamic network. This review focuses on and explores how acetylation and deacetylation affect the activity of key signalling pathways, transcription factors and metabolic enzymes related to metabolic reprogramming, thereby altering the metabolic patterns of cancer cells to better adapt to the needs of rapid proliferation and survival. The PI3K/AKT/mTOR signalling pathway plays a central role in regulating cellular metabolism. KAT6A acetylates histone H3, activating PIK3CA transcription, thereby enhancing the PI3K/AKT signalling pathway and promoting glioblastoma development.^36^ P53 inhibits glycolysis, and in most cases, SIRT1 mediates p53 deacetylation, reducing its transcriptional activity and thereby suppressing tumour cell apoptosis.^151^ PFK is the second rate‐limiting enzyme in glycolysis. KAT5 mediates PFKP acetylation, leading to its membrane translocation, subsequently activating PFK1 and increasing GLUT1 expression, thereby promoting glycolysis and tumour cell proliferation.^222^ Currently, extensive research has focused on the regulatory effects of acetylation/deacetylation on signalling pathways and transcription factors. In contrast, few studies have investigated the metabolic enzymes involved in acetylation/deacetylation. In the complex environment of cancer, the precise role of acetylation may change depending on the type of substrate involved, the tissue type, the specific stage of the tumour or the metabolic conditions. For example, after the deacetylation of the transcription factor NRF2 by the deacetylase SIRT1, the transcriptional activity of NRF2 is activated.^200^ Conversely, after the acetylation of NRF2 by the acetyltransferase CBP, the transcriptional activity of NRF2 is also activated.^201^ This suggests that different acetylation sites may exert distinct regulatory effects on the same protein. Hence, continued in‐depth research is necessary to elucidate the specific role of acetylation modifications in tumour metabolism. This study focused mainly on a few specific enzymes and proteins, while the tumour metabolism network is intricate and involves many metabolic enzymes and signalling pathways. Therefore, research on other potential acetylation sites and their mechanisms remains insufficient. In the future, we need to continue exploring and validating more acetylation sites of metabolic enzymes and signalling pathways and their specific effects on tumour metabolism and growth. Additionally, attention should be given to the interaction between acetylation and other epigenetic regulatory mechanisms (such as methylation, phosphorylation, etc.) to further reveal the regulatory network of tumour metabolic reprogramming.

Given the central role of acetylation and deacetylation in cancer biology, therapies targeting these processes have potential clinical applications, particularly by inhibiting or activating enzymes involved in acetylation. HDAC inhibitors are among the most mature therapeutic strategies for targeting acetylation. Several HDAC inhibitors have been approved or are in clinical trial stages. Targeting sirtuins in cancer may have therapeutic effects by preventing cancer cells from surviving and maintaining metabolic flexibility under metabolic stress (e.g., hypoxia or nutrient deprivation). HDAC inhibitors are often used in combination with other therapies, such as chemotherapy, immunotherapy and targeted therapies (e.g., kinase inhibitors), to enhance anti‐cancer effects. For example, HDAC inhibitors could improve the effectiveness of immune checkpoint inhibitors through their ability to alter the tumour microenvironment. In addition to histones, the acetylation of non‐histone proteins such as metabolic enzymes and transcription factors also provides potential therapeutic targets. Drugs that regulate the acetylation status of these proteins can directly disrupt cancer metabolism.

The clinical application of HDAC inhibitors still faces multiple challenges. First, HDAC inhibitors often target multiple HDAC subtypes, which may lead to toxic reactions.^295^ Current research is focused on developing more selective inhibitors that target specific HDACs or sirtuins to reduce side effects while maintaining therapeutic efficacy.^296^ Second, cancer cells have metabolic flexibility, and regulating a particular metabolic pathway through acetylation may activate other compensatory mechanisms.^297^ Combined therapies that target multiple metabolic pathways or processes simultaneously may help overcome this challenge. Finally, identifying biomarkers associated with changes in acetylation in tumours can aid in personalised treatment by identifying patients most likely to respond to HDAC inhibitors or other acetylation‐regulating drugs.

In summary, acetylation and deacetylation are considered critical regulators of metabolic reprogramming in tumour cells. Targeting acetyltransferases and deacetylases, particularly HDACs, has shown promising potential in cancer therapy. People also need to further optimise these therapeutic approaches to maximise their clinical benefits and pioneer new strategies for cancer treatment.

## AUTHOR CONTRIBUTIONS

**Cuicui Wang and Xiaoxin Ma**: conceived the review. **Cuicui Wang**: wrote the first version of the manuscript. **Xiaoxin Ma**: revised the manuscript. All the authors approved the final version of the manuscript.

## CONFLICT OF INTEREST STATEMENT

The authors declare no conflicts of interest.

### ETHICS STATEMENT

Not applicable.

## Supporting information



Supporting Information

## Data Availability

All the data obtained and/or analysed during the current study were available from the corresponding authors on reasonable request.
